# A New Perspective on *Listeria monocytogenes* Evolution

**DOI:** 10.1371/journal.ppat.1000146

**Published:** 2008-09-05

**Authors:** Marie Ragon, Thierry Wirth, Florian Hollandt, Rachel Lavenir, Marc Lecuit, Alban Le Monnier, Sylvain Brisse

**Affiliations:** 1 Institut Pasteur, Laboratoire des Listeria, Paris, France; 2 Institut Pasteur, Centre National de Référence des Listeria and World Health Organization Collaborating Centre for Foodborne Listeriosis, Paris, France; 3 Ecole Pratique des Hautes Etudes, Muséum National d'Histoire Naturelle, Department of Systematics and Evolution, Paris, France; 4 Institut Pasteur, Genotyping of Pathogens and Public Health Platform (PF8), Paris, France; 5 Institut Pasteur, Microbes and Host Barriers Group, Paris, France; 6 Inserm, Avenir U604, Paris, France; 7 Université Paris Descartes, Hôpital Necker-Enfants malades, Service des Maladies Infectieuses et Tropicales, Centre d'Infectiologie Necker-Pasteur, Paris, France; SUNY at Stony Brook, United States of America

## Abstract

*Listeria monocytogenes* is a model organism for cellular microbiology and host–pathogen interaction studies and an important food-borne pathogen widespread in the environment, thus representing an attractive model to study the evolution of virulence. The phylogenetic structure of *L. monocytogenes* was determined by sequencing internal portions of seven housekeeping genes (3,288 nucleotides) in 360 representative isolates. Fifty-eight of the 126 disclosed sequence types were grouped into seven well-demarcated clonal complexes (clones) that comprised almost 75% of clinical isolates. Each clone had a unique or dominant serotype (4b for clones 1, 2 and 4, 1/2b for clones 3 and 5, 1/2a for clone 7, and 1/2c for clone 9), with no association of clones with clinical forms of human listeriosis. Homologous recombination was extremely limited (r/m<1 for nucleotides), implying long-term genetic stability of multilocus genotypes over time. Bayesian analysis based on 438 SNPs recovered the three previously defined lineages, plus one unclassified isolate of mixed ancestry. The phylogenetic distribution of serotypes indicated that serotype 4b evolved once from 1/2b, the likely ancestral serotype of lineage I. Serotype 1/2c derived once from 1/2a, with reference strain EGDe (1/2a) likely representing an intermediate evolutionary state. In contrast to housekeeping genes, the virulence factor internalin (InlA) evolved by localized recombination resulting in a mosaic pattern, with convergent evolution indicative of natural selection towards a truncation of InlA protein. This work provides a reference evolutionary framework for future studies on *L. monocytogenes* epidemiology, ecology, and virulence.

## Author Summary

*Listeria monocytogenes* is a pathogen transmitted through contaminated food and is responsible for severe infections, including meningitis and abortion in animals and humans. It is known that many distinct strains of this pathogen exist, and that they differ in their virulence and epidemic potential. Unfortunately, there is currently no standard definition of strains and no comprehensive overview of their evolution. To tackle these serious limitations to the control of listeriosis and to improve knowledge of how virulence evolves, we characterized a large collection of isolates with sequence-based genotyping methods. We were thus able to identify precisely the most prevalent clones of *L. monocytogenes*, i.e., groups of isolates that descend from a single ancestral bacterium, which can now be characterized further for diagnostic purposes and determination of their precise ecology and virulence potential. We also determined how these clones evolved from their common ancestor and the evolutionary history by which they acquired their phenotypic characteristics, such as antigenic structures. Finally, we show that some particular strains tend to lose a virulence factor that plays a crucial role in infection in humans. This is a rare example of evolution towards reduced virulence of pathogens, and the discovery of the selective forces behind this phenomenon may have important epidemiological and biological implications.

## Introduction

The opportunistic pathogen *Listeria monocytogenes* causes life-threatening infections in animal and in human populations at risk. This facultative intracellular bacterium is widespread in the environment and infections occur through ingestion of contaminated food [1],[2]. Although the species *L. monocytogenes* has long been known to be genetically diverse [3], with strains showing differences in their virulence potential [4]–[7], detailed knowledge of strain diversity and evolution is still lacking.

Several methods have been used to differentiate *L. monocytogenes* strains [8]. The *Listeria* serotyping scheme [9] based on somatic (O) and flagellar (H) antigens currently represents a common language for *L. monocytogenes* isolate typing and investigations into the ecological distribution, epidemiology and virulence of strains. Unfortunately, serotyping discriminates only 13 serotypes, many of which are known to represent genetically diverse groups of strains, and only four serotypes (1/2a, 1/2b, 1/2c, and 4b) cause almost all cases of listeriosis in humans [1]. Given its higher discriminatory power, pulsed-field gel electrophoresis (PFGE) is considered accurate for epidemiological investigations and of help for surveillance and control of listeriosis [10],[11], but fingerprint-based methods such as PFGE or ribotyping [12] are difficult to standardize. Hence, inter-laboratory comparisons necessitate considerable harmonization [13], which limits knowledge at the global scale. In addition, these widely used methods provide only limited information on the phylogenetic relationships among strains, which is a serious limitation to understand the evolution of important phenotypic traits such as virulence. Sequence-based or SNP-based approaches appear as promising tools for strain typing and phylogeny in *L. monocytogenes* [14]–[17]. Multilocus sequence typing (MLST) [18]–[20] can accurately define the clonal framework of bacterial species. MLST has been shown to discriminate among *L. monocytogenes* isolates [14],[21],[22], but has not yet been applied on a large scale, and an overview of the clonal structure of *L. monocytogenes* is currently not available. The molecular factors that determine ecological differences among strains are also poorly understood.

One salient feature of the population structure of *L. monocytogenes* is the distinction of three phylogenetic lineages. Initially, two major lineages were distinguished, mainly based on multilocus enzyme electrophoresis and PFGE [3],[10],[12],[23],[24], with a third lineage being subsequently recognized based on virulence gene variation, ribotyping and DNA arrays [25]–[28]. Lineage I includes isolates of serotypes 4b, 1/2b, 3b, 4d and 4e, whereas lineage II includes serotypes 1/2a, 1/2c, 3a and 3c. Lineage III contains serotypes 4a and 4c, as well as serotype 4b as was recently discovered [27]. The relative virulence and contribution of the three lineages and their serotypes to food contamination and clinical burden is subject of debate [3], [26], [27], [29]–[32]. As each lineage is genetically heterogeneous, a precise delineation of *L. monocytogenes* clones is needed to determine which ones mostly contribute to human or animal infection [16],[33],[34], and this knowledge would set a landmark for further studies on the biological characteristics of the clones and the evolution of molecular mechanisms by which they cause disease [35].

Several virulence genes play an important role in the virulence of *L. monocytogenes* strains [36],[37]. Internalin (InlA) is a surface protein that mediates the entry of *L. monocytogenes* into various non-phagocytic human eukaryotic cells expressing its receptor E-cadherin [38],[39] and plays a key role in the crossing of the intestinal barrier, enabling the bacterium to reach the host bloodstream [40]. Almost all isolates causing listeriosis in humans express a full-length functional InlA, whereas isolates expressing a truncated form are frequently found in food items and the environment and are associated with a lower virulence potential [5]. Currently, the ecological factors that drive the evolution of these apparently attenuated strains are unknown. Evolution of virulence would be best understood by mapping the variation of virulence genes such as *inlA*, onto the phylogenetic framework of the genomes in which they are presently distributed.

The aims of this study were to provide a robust phylogenetic framework based on MLST analysis of a highly diverse isolate collection and determine (i) the population structure of *L. monocytogenes*; (ii) the evolutionary origin and stability of serotypes; and (iii) the patterns of variation of the virulence gene *inlA* with respect to the evolution of the core genome.

## Materials and Methods

### Bacterial isolates

A total of 360 *Listeria monocytogenes* and four *L. innocua* isolates were selected from the collections of the French National Reference Centre for *Listeria* and the WHO Collaborative Centre for foodborne listeriosis (**Table S1**). These 360 *L. monocytogenes* isolates were subdivided in three subsets, each being included in order to address specific questions: (i) a diversity subset of 171 isolates, which included representative isolates of the distinct *L. monocytogenes* serotypes, atypical strains from lineage III, isolates that caused major epidemics throughout the world, strains for which the complete genome sequence is available, 75 historical strains collected from 1924 to 1966 and belonging to H.P.R. Seeliger *Listeria* Culture Collection (Würzburg, Germany), isolates from the environment, food or animals, and research strains from several countries used in previous studies involving the Institut Pasteur *Listeria* laboratory (**Table S1**); (ii) 126 isolates selected from maternal-fetal cases, collected prospectively and exhaustively from 1987 to 2005 (i.e., 5 to 10 epidemiologically non-related isolates randomly selected per year), and which were included to probe the temporal dynamics of clone prevalence (‘MF chronological’ subset in **Table S1**); and (iii) 63 isolates from year 2000, including 25 from bacteremia, 20 from central nervous system (CNS) infection, and 18 from maternal-fetal infection, which were included to investigate the possible association of specific clones with clinical forms (subset ‘Human clinical, 2000’ in **Table S1**).

Isolates were identified as *L. monocytogenes* using API *Listeria* strips (BioMerieux, La Balme Les Grottes, France). Identification was confirmed and subdivided into serotypes by classical serotyping [9], which distinguishes 13 serotypes, and multiplex PCR [41], which groups *L. monocytogenes* isolates into four major groups (IIA, IIB, IIC et IVB) corresponding to groups of serotypes (**Table S1**).

### Multilocus Sequence Typing

The MLST scheme used to characterize *Listeria* strains is based on the sequence analysis of the following seven housekeeping genes: *acbZ* (ABC transporter), *bglA* (beta-glucosidase), *cat* (catalase), *dapE* (Succinyl diaminopimelate desuccinylase), *dat* (D-amino acid aminotransferase), *ldh* (lactate deshydrogenase), and *lhkA* (histidine kinase). This MLST scheme was adapted from the MLST system proposed by Salcedo and colleagues [14], with the following modifications. First, the template for gene *ldh* was extended from 354 to 453 nucleotides, thus improving strain discrimination. Second, gene templates were shortened because the extremities of the previous templates correspond to parts of the PCR primer sequences, thus possibly not corresponding totally to the genomic sequence of the isolates analyzed. Third, we incorporated universal sequencing tails to the PCR primers (**Table 1**), which allows to sequence PCR fragments of all genes using only two primers. DNA extraction was performed by the boiling method [41]. The PCR amplification conditions were as follows: an initial cycle of 94°C for 4 min; 25 amplification cycles, each consisting of 94°C for 30 s, 52°C for 30 s (except for *bglA* which has an annealing temperature of 45°C), and 72°C for 2 min; and a final incubation at 72°C for 10 min. The PCR products were purified by ultrafiltration (Millipore, France) and were sequenced on both strands with Big Dye v.1.1 chemistry on an ABI3730XL sequencer (Applied BioSystems).

**Table 1:** PCR and sequencing primers used.

Locus	Putative function of gene	Forward primer	Reverse primer	Location^a^	Annealing temperature (°C)
*abcZ*	ABC transporter	GTTTTCCCAGTCACGACGTTGTATCGCTGCTGCCACTTTTATCCA	TTGTGAGCGGATAACAATTTCTCAAGGTCGCCGTTTAGAG	2,828,236 to 2,830,008	52
*bglA*	Beta glucosidase	GTTTTCCCAGTCACGACGTTGTAGCCGACTTTTTATGGGGTGGAG	TTGTGAGCGGATAACAATTTCCGATTAAATACGGTGCGGACATA	343,221 to 344,636	45
*cat*	Catalase	GTTTTCCCAGTCACGACGTTGTAATTGGCGCATTTTGATAGAGA	TTGTGAGCGGATAACAATTTCAGATTGACGATTCCTGCTTTTG	2,871,318 to 2,872,784	52
*dapE*	Succinyl diaminopimelate desuccinylase	GTTTTCCCAGTCACGACGTTGTACGACTAATGGGCATGAAGAACAAG	TTGTGAGCGGATAACAATTTCATCGAACTATGGGCATTTTTACC	287,853 to 288,992	52
*dat*	D-amino acid aminotransferase	GTTTTCCCAGTCACGACGTTGTAGAAAGAGAAGATGCCACAGTTGA	TTGTGAGCGGATAACAATTTCTGCGTCCATAATACACCATCTTT	1,661,588 to 1,662,457	52
*ldh*	L-lactate dehydrogenase	GTTTTCCCAGTCACGACGTTGTAGTATGATTGACATAGATAAAGA	TTGTGAGCGGATAACAATTTCTATAAATGTCGTTCATACCAT	214,486 to 215,427	50
*lhkA*	Histidine kinase	GTTTTCCCAGTCACGACGTTGTAAGAATGCCAACGACGAAACC	TTGTGAGCGGATAACAATTTCTGGGAAACATCAGCAATAAAC	1,538,498 to 1,539,937	52
*Sequencing primers for above genes*		Forward: GTT TTC CCA GTC ACG ACG TTG T	Reverse: TTG TGA GCG GAT AAC AAT TTC		
*inlA*	Internalin	CGGATGCAGGAGAAAATCC	CTTTCACACTATCCTCTCC	454,534 to 456,936	55
*inlA internal sequencing primers*		F1: GATATAACTCCACTTGGG	R1: GCTCTAAGTTAGTGAGTGCG		
*inlA internal sequencing primers*		F2: GTGGACGGCAAAGAAAC	R2: GAGATGTTGTTACACCGTC		

a Positions correspond to complete genome sequence of strain EGDe (NC003210).

### *inlA* gene sequencing

The 2,400 bp long *inlA* gene was sequenced from 157 isolates (**Table S1**) representing the clonal diversity of *L. monocytogenes* (see below). DNA extraction was performed with the Wizard® kit (Promega Corporation, USA). The PCR amplification conditions were as follows: an initial cycle at 94°C for 5 min; 35 amplification cycles, each consisting of 94°C for 30 s, 55.2°C for 30 s, and 72°C for 1 min 30; and a final incubation at 72°C for 10 min. We used external primers for amplification and internal primers for sequencing (**Table 1**), which was performed as described above.

### Data analysis

For each MLST locus, an allele number was given to each distinct sequence variant, and a distinct sequence type (ST) number was attributed to each distinct combination of alleles at the seven genes. Numbers were initially based on highest frequency for the frequent alleles and STs, and were subsequently incremented arbitrarily. In order to define the relationships among strains at the microevolutionary level, we performed allelic profile-based comparisons using a minimum spanning tree (MST) analysis with the BioNumerics v5.10 software (Applied-Maths, Sint Maartens-Latem, Belgium). MST analysis links profiles so that the sum of the distances (number of distinct alleles between two STs) is minimized [42]. Strains were grouped into clonal complexes (clonal families), defined as groups of profiles differing by no more than one gene from at least one other profile of the group [19]. Accordingly, singletons were defined as STs having at least two allelic mismatches with all other STs.

Neighbor-joining tree analysis was performed using MEGA v4 [43] or SplitsTree v4b06 [44]. Calculations of recombination tests were performed using RDP3 [45]. Nucleotide diversity indices were calculated using DNAsp v4 [46]. ClonalFrame analysis [47] was performed with 50,000 burn-in iterations and 100,000 subsequent iterations.

To test for phylogenetic congruence among genes, one strain of all 39 STs with allelic mismatch distance >0.65 was used in order to exclude the expected congruence among genes at small evolutionary scale due to common clonal descent, as proposed previously [48]. Neighbor-joining trees were generated using PAUP* v4 [49] for each gene individually and for the concatenated sequence of the seven genes. For each gene, the differences in log likelihood (Δ−ln L) were computed using PAUP* between the tree for that gene and the trees constructed using the other genes, with branch lengths optimized [50]. These differences were compared to those obtained for 200 randomly generated trees.

The relative contribution of recombination and mutation on the short term was calculated using software MultiLocus Analyzer (Brisse, unpublished) and the simplest implementation of the clonal diversification method [51],[52]. For each pair of allelic profiles that are closely related, the number of nucleotide changes between the alleles that differ is counted. A single nucleotide difference is considered to be likely caused by mutation, whereas more than one mutation in the same gene portion is considered to derive from recombination, as it is considered unlikely that two mutations would occur on the same gene while the other genes remain identical. No correction was made for single nucleotide differences possibly introduced by recombination.

We used the linkage model in Structure [53] to identify groups with distinct allele frequencies [53]. This procedure assigns a probability of ancestry for each polymorphic nucleotide for a given number of groups, *K*, and also estimates *q*, the combined probability of ancestry from each of the *K* groups for each individual isolate. We chose three groups for this report because repeated analyses (200,000 iterations, following a burn-in period of 100,000 iterations) with *K* between 1 and 10 showed that the model probability increased dramatically between *K* = 2 and *K* = 3 and only slowly thereafter.

The population recombination rate was estimated by a composite-likelihood method with LDhat [54]. LDhat employs a parametric approach, based on the neutral coalescent, to estimate the scaled parameter 2*N*_e_ *r* where *N_e_* is the effective population size, and *r* is the rate at which recombination events separate adjacent nucleotides. The crossing-over model L was used for the analysis of biallelic sites.

We also tested for presence of positively selected sites using the software omegaMap [55]. This program applies a coalescence-based Bayesian strategy that co-estimates the rate of synonymous vs. non-synonymous substitions ω and the population recombination rate ρ, thus circumventing the high rate of false positives arising from incongruent phylogenies [56]. The following prior distributions were used for the analyses: μ, κ and Φ_indel_: improper inverse, ω: inverse with range 0.0001–10, ρ: inverse with range 0.01–10. The variable block model was chosen for both ω and ρ, with block sizes of 10 and 30, respectively. We created 10 subsets of 50 randomly drawn *inlA* sequences each, and analyzed each subset with 50,000 iterations and 10 reorderings. The first 20,000 sequences were discarded as burn-in period.

### Nucleotide sequences

Sequences generated in this study are available at www.pasteur.fr/mlst for the seven MLST genes. *inlA* sequences have been deposited in GenBank/EMBL/DDBJ databases under the accession numbers FM178779 to FM178796 and FM179771 to FM179785. Alleles of the seven MLST genes were deposited under the accession numbers FM180227 to FM180445.

## Results

### The majority of clinical isolates of *L. monocytogenes* belong to seven distinct clones

**Figure 1:**
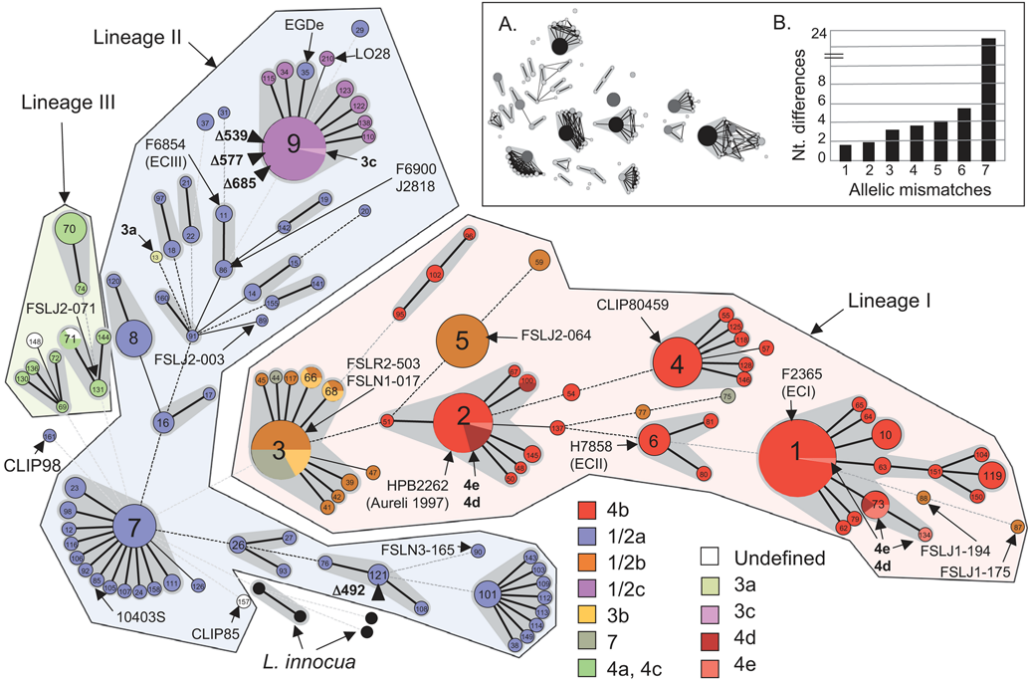
Minimum spanning tree analysis of 360 *L. monocytogenes* and four *L. innocua* strains based on MLST data. Each circle corresponds to a sequence type (ST). Grey zones surround STs that belong to the same clonal complex (CC; 24 CCs are visible in total). ST numbers are given inside the circles and are enlarged for the central genotypes that define the major CCs (e.g., ST9 defines the central genotype of CC9). The three major lineages are highlighted by polygons. Four *L. innocua* sequence types are also represented (black circles). The lines between STs indicate inferred phylogenetic relationships and are represented as bold, plain, discontinuous and light discontinuous depending on the number of allelic mismatches between profiles (1, 2, 3 and 4 or more, respectively); note that discontinuous links are only indicative, as alternative links with equal weight may exist. There were no common alleles between the three major lineages, *L. innocua*, ST161 (CLIP98) and ST157 (CLIP85); they are arbitrarily linked through ST7 by default. Circles and sectors were colored based on serotyping data according to the provided legend; in addition, rare serotypes (3a, 3c, 4d, 4e) are indicated directly on the Figure. Note that for simplicity, the serotype of strains that were serotyped by the PCR method (Table S1) was equated to the most frequent serotype of each PCR group (e.g., 1/2a for PCR group IIA). STs in which truncated forms of InlA were found are indicated by a black triangle, with the position of the premature stop codons given after letter Δ. The ST of reference genome strains is indicated. The positioning of H7858 (ECII) is based on 6 genes only, as gene *dat* is incomplete. Inset A. Crosslinks corresponding to one or two allelic mismatches are indicated. Note the absence of links among major clonal complexes, indicative of their neat demarcation. Circles were colored by grey levels according to the number of isolates. Inset B. Correlation between the number of allelic mismatches (number of distinct alleles between MLST profiles) and the average number of nucleotide differences at distinct alleles. Note the regular positive trend, which indicates that *L. monocytogenes* genotypes diverge predominantly by a mutational process [81]. Allelic mismatch values of 7 correspond mostly to inter-lineages comparisons.

The seven gene portions, sequenced in the 360 *L. monocytogenes* isolates, harbored a total of 438 polymorphisms (13.3%; range 7.01%–17.7% per gene) consisting in bi-allelic (404 sites), tri-allelic (32 sites) or four-allelic (2 sites) single nucleotide polymorphisms (SNPs). The average nucleotide diversity π was 2.91%, ranging from 1.18% to 5.98% per gene (**Table 2**). The GC% observed in all alleles ranged from 36.5% to 43.3%, consistent with the 39% value observed across the entire *L. monocytogenes* EGDe genome [57]. The 126 resulting allelic profiles (or sequence types, STs) were distributed into twenty-three clonal complexes (CC) and 22 singletons (**Figure 1**). Five CCs (CC2 to CC4, CC7 and CC9) consisted of a central prevalent genotype associated with several much less-frequent single locus variants (SLVs). CC1 was slightly more diverse, as its central genotype had two SLVs that themselves were associated to other variants. ST5 stood out among all singletons by its high frequency. Each of these CCs and singletons is likely to have descended from a single ancestral bacterium, i.e. corresponds to a clone. Remarkably, the seven above-mentioned CCs were well demarcated, as they differed by at least four genes out of seven among themselves (with the exception of CC2 and CC3, with three mismatches between one pair of STs) and by at least three mismatches from all other STs (**Figure 1, inset A**). Together, these seven clones comprised 58 (47%) STs and 245 (69%) isolates, and included 73% of the 252 recent (after 1987) clinical isolates. Five of these clones belonged to lineage I (see below) and comprised 177 of 203 (87%) isolates of this lineage. Other frequent clones were CC6, CC8 and CC101, together representing 32 (9%) additional isolates. Reference strains of large outbreaks and genome sequencing project strains were mapped on the disclosed MLST diversity (**Figure 1** **;** **Table S1**); for example, ST1, ST6 and ST11 include reference strains of epidemic clones I, II and III [33],[34], respectively.

**Table 2:** Polymorphism of seven housekeeping protein-coding genes among *L. monocytogenes* isolates.

Gene	Template size	No. alleles	No. (%) polymorphic sites	Ks	Ka	Ka/Ks	π
*abcZ*	537	20	51 (9.49)	0.09624	0.00139	0.014	0.0208
*bglA*	399	18	28 (7.01)	0.05412	0.00058	0.0107	0.0118
*cat*	486	29	47 (9.67)	0.09882	0.00287	0.029	0.0221
*dapE*	462	26	82 (17.7)	0.15317	0.00894	0.058	0.0358
*dat*	471	16	70 (14.9)	0.31024	0.014	0.045	0.0598
*ldh*	453	71	79 (17.4)	0.10767	0.00235	0.0218	0.0232
*lhkA*	480	12	81 (16.9)	0.16468	0.00437	0.0265	0.0289
Concatenate, 353 strains	3,288	121	438 (13.3)	0.12885	0.0049	0.038	0.0291
Concatenate, Lineage I (199 strains)	3,288	48	53 (1.61)	0.0124	0.00067	0.054	0.0033
Concatenate, Lineage II (133 strains)	3,288	61	143 (4.35)	0.02481	0.00076	0.0306	0.0061
Concatenate, Lineage III (19 strains)	3,288	11	156 (4.74)	0.04896	0.00258	0.0527	0.0125

Ks: No. of synonymous changes per synonymous site. Ka: No. of non-synonymous changes per non-synonymous site.

π: nucleotide diversity.

Remarkably, most isolates within a given clone had the same serotype, or a restricted set of serotypes. CC1 and CC2 were dominated by isolates of serotype 4b, and included all isolates of serotypes 4d and 4e. CC3 comprised a large proportion (19/42, 45%) of isolates of serotype 1/2b, and included all isolates of serotypes 3b, and all but one (ST75) isolates of serotype 7. These results suggest that serotypes 4d and 4e each derived at least twice from 4b ancestors, consistent with previous data [16],[28], whereas isolates of serotypes 3b and 7 (excepted ST75) may be regarded as serotypic variants of serotype 1/2b CC3 isolates. CC4 (serotype 4b), ST5 (1/2b), CC6 (4b), CC7 (1/2a), CC101 (1/2a) and CC102 (4b) were each homogeneous with respect to serotype. Finally, CC9 included all isolates of serotype 1/2c, indicating that this serotype is genetically homogeneous. Notably, the virulent strain EGDe of serotype 1/2a also fell into CC9. EGDe only differs from ST9 (1/2c) by *dapE* (allele dapE-20 instead of dapE-4 in ST9), while it differs from all other 1/2a strains by several genes. CC9 also comprised the only included isolate of serotype 3c.

Among isolates from human cases of listeriosis, we sought to determine the possible association between clones and clinical sources of the isolates. To eliminate the possible effect of the temporal variation (see below), we compared *L. monocytogenes* isolates from a single year (year 2000) and the three major clinical presentations in humans: bacteremia (n = 25), CNS infections (n = 20), and maternal-fetal infections (n = 18). These isolates corresponded to 28 STs, distributed into 7 CCs and 13 singletons (**Table S1**). There was no association of particular CCs or ST with clinical presentation: the 11 STs with more than one isolate were encountered in at least two clinical sources, and isolates from prevalent CCs or STs were equally isolated from the three clinical forms (**Table S1**).

Possible trends in the relative prevalence of CCs over time were investigated based on 126 isolates from maternal-fetal cases of listeriosis, collected from 1987 to 2005 (**Table S1**). These isolates (**Table S1**) fell into 43 STs and were grouped into 7 CCs and 14 singletons. Four CCs (CC1 to CC4) and two singletons (ST5 and ST9) comprised more than 10 isolates. Numbers of isolates of each of these clones over the 19 year period showed distinct patterns of temporal dynamics: while CC1 (4b) and ST9 (1/2c) were sampled equally over the entire period, CC3 (1/2b-3b-7) shows a clear decrease in prevalence (16 isolates before 1995, 2 isolates after; Chi2 p<0.001). In contrast, ST5 (1/2b) was isolated only once before 1997 but 12 times in the second period (p = 0.02). Similarly, CC2 (4b) showed an apparent increase in prevalence (2 vs. 9, p = 0.034).

### Homologous recombination is rare in *Listeria monocytogenes*

Divergence among genotypes appeared to be mainly driven by the progressive accumulation of mutations over time, as strains diverge from their common ancestor (**Figure 1, inset B**). Congruence among the seven individual gene phylogenies obtained for the distantly related STs [48] was statistically significant (p<0.005), as assessed by the likelihood method [50]. Similarly, the short-term contribution of recombination to genotypic diversity was modest, as *L. monocytogenes* alleles are five times more likely to change by mutation than by recombination (*r/m* = 0.197). In addition, the *r/m* rate for nucleotides was 0.59, indicating that nucleotides are approximately twice more likely to change by mutation than by recombination. As an independent approach, the composite likelihood of *r/m* [54] on the concatenated sequence of the seven genes was 0.62 for lineage I, 0.47 for lineage II and 0 for lineage III. *r/m* values of some of the observed housekeeping genes exceeded 1, but lacked statistical significance (**Table 3**). Consistently, *r/m* was 0.81 as estimated using ClonalFrame [47].

**Table 3:** Comparison of mutation rates (*μ*) and recombination rates (r) per base.

Group	Θ			*ρ*			r/*μ*		
	I	II	III	I	II	III	I	II	III
ldh	0.00519	0.01553	0.00629	0.15011	0.01325 **	0.00625	28.923	0.8529 **	0.99364
dapE	0.00295	0.1342	0.1762	0	0	0	0	0	0
bglA	0.00342	0.00790	0.00499	0.01013 **	0.01000 **	0 **	0.0296 **	0.7900 **	0 **
lhkA	0.00121	0.00121	0.04512	0.01042	0.05208	0	8.609	43.044	0
dat	0.0165	0.00248	0.00422	0	0	0	0	0	0
abcZ	0.00290	0.00437	0.01179	0	0.00372 **	0.00372	0	0.8523 **	0.6165
cat	0.00200	0.01273	0.00527	0.00823	0	0	4.1152	0	0
Concat	0.00296	0.00834	0.01352	0.00182 **	0.00395 **	0	0.6165 **	0.4741 **	0

a Concat., concatenated data set. Values for rho (ρ) were obtained by dividing the per-locus recombination rate estimate from LDhat by the sequence length.

**: Estimates that are significant at the 5% level. Θ and *ρ* correspond to the population estimates of mutation and recombination rates, respectively.

**Figure 2:**
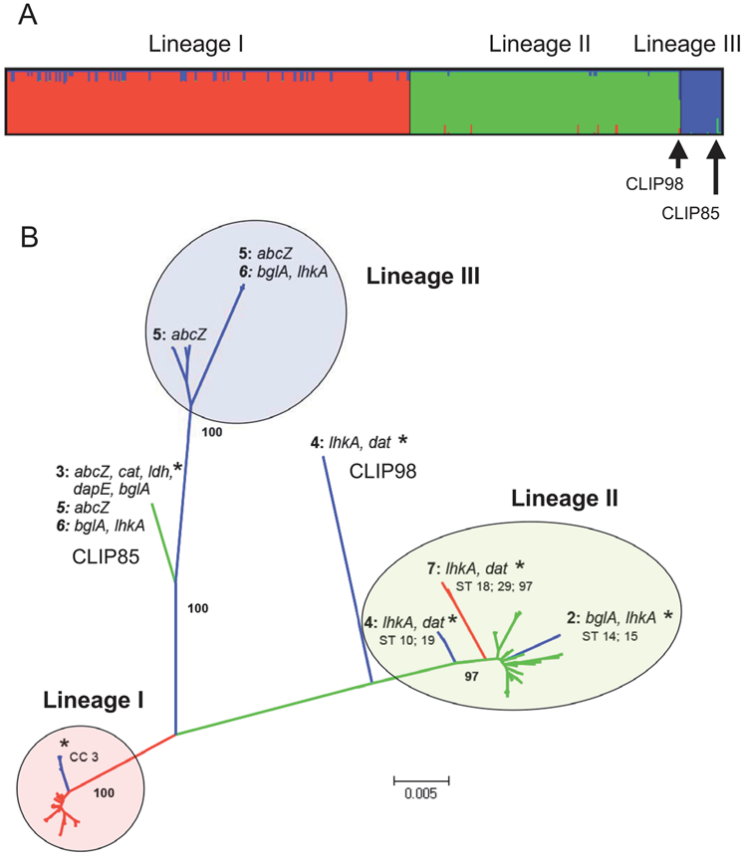
Homologous recombination is rare, but distorts phylogenetic reconstruction. A) Proportions of ancestry in seven housekeeping genes of *L. monocytogenes* strains from three ancestral populations as inferred by the linkage model of structure. This plot shows one vertical line for each isolate in which the proportions of ancestry from the three sources are color-coded. For example, strain CLIP85 was inferred as having mixed ancestry, with approximately 75% of nucleotides originated from lineage III (blue), whereas 25% of them were inferred to have an origin in lineage II (green). A number of strains from lineage I have a small proportion of nucleotides with ancestry in lineage III, while strains of lineage II had some nucleotides from lineages I (red) or III (blue). The case of CLIP98 is particular, as it was inferred as deriving from lineage II by imports mainly from *L. innocua* (see text). B) Neighbor-joining phylogenetic analysis of concatenated housekeeping gene sequences, using the Tamura-Nei+G+I model. The three major *L. monocytogenes* lineages are recognized. Together, they included all strains except CLIP85 and CLIP98. Bootstrap support of lineages is given at corresponding branches. An asterisk (*) marks strains that were recognized by structure to contain a fraction of nucleotides imported from another ancestral population, with corresponding branches colored according to the source of the recombined nucleotides. Recombination events detected independently using with RDP3 are numbered from 1 to 7 (referring to Table S2), and the involved genes are indicated.

In order to determine which lineages underwent recombination events that left a detectable footprint in extant strains, the nucleotide polymorphisms within the seven gene fragments were analyzed with structure [53],[58], a Bayesian method that attempts to identify the ancestral sources of nucleotides. The ancestry of each isolate can be estimated as the summed probabilities of derivation from each ancestral group over all polymorphic nucleotides. structure recognized three clusters of strains within *L. monocytogenes*, which were largely homogeneous in terms of their ancestral sources of polymorphism (**Figure 2A**). However, a number of isolates are likely to have a mixed origin (**Figure 2A**), and this was confirmed statistically using RDP3 on the concatenated sequences (**Table S2**).

### Phylogenetic structure of *L. monocytogenes* and evolutionary origin of serotypes

**Figure 3:**
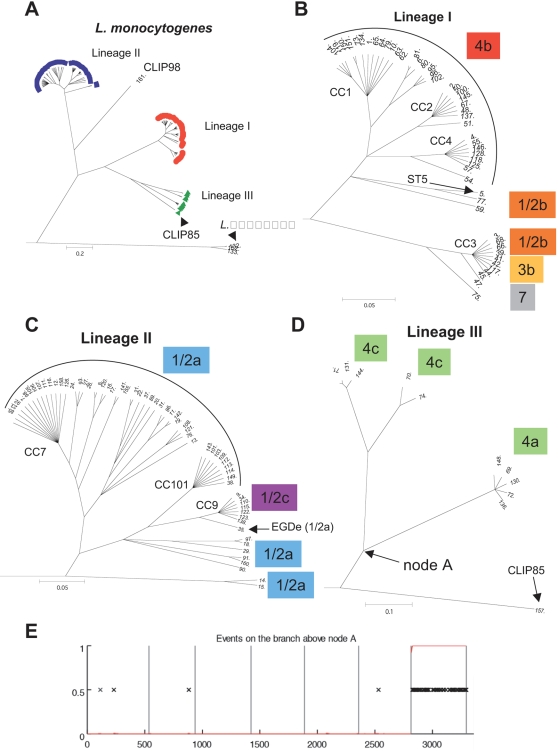
Phylogeny obtained with ClonalFrame and serotype relationships. A) 50% consensus tree obtained after 100,000 iterations (after 50,000 burn-in) for the 130 distinct *Listeria* STs. *L. monocytogenes* appears monophyletic, with three distinct lineages and one strain (CLIP98) considered as a fourth lineage. Note the close association of CLIP85 with other lineage III strains. B) Detailed view of the inferred relationships within lineage I. Note the monophyly of serotype 4b. C) Detailed view of the inferred relationships within lineage II. Note that all strains of serotype 1/2c (CC9) are nested inside the diversity of 1/2a, and that serotype 1/2c seems to have evolved from 1/2a just after the split from strain EDGe. D) Detailed view of the inferred relationships within lineage III. E) Events on the branch that separates CLIP85 (ST157) from the rest of lineage III. Note the high number of nucleotide changes in *lhkA* (seventh gene), inferred by clonalFrame to correspond to a single recombination event.

Because recombination events, even if they are rare, can strongly distort phylogenetic reconstruction, we took into account potential recombination events using ClonalFrame (**Figure 3**). The majority-rule consensus tree revealed three major branches, which could be equated to the three currently recognized *L. monocytogenes* lineages I, II and III, as deduced from serotyping data and inclusion of reference strains. In particular, strains with serotypes 4b and 1/2b fell into lineage I, serotypes 1/2a and 1/2c were associated with lineage II, whereas serotypes 4a and 4c belonged to lineage III. The neighbor-joining (NJ) method (**Figure 2**) retained the three major lineages, which were also consistent with the three major groups revealed by Structure. However, the obtained branching pattern was conspicuously distinct for those isolates that underwent recombination events. The most conspicuous example was isolate CLIP85, which was clearly associated with lineage III (**Figure 3**), but not in the NJ tree (**Figure 2B**). This difference could be attributed to horizontal transfer of *lhkA* from lineage II into CLIP85, as detected with high probability by ClonalFrame (**Figure 3E**). Likewise, strains that were inferred to have mixed ancestries (**Figure 2A**) were placed at the tip of relatively longer branches on the NJ tree (**Figure 2B**) than on the tree derived from ClonalFrame tree (**Figure 3**).

One exceptional isolate, CLIP98 (serotype 1/2a) isolated from a human blood infection in Canada, was placed at the tip of a long branch, thus representing an apparent fourth lineage. Individual gene genealogies based on the neighbor-joining method also placed CLIP98 outside the three lineages, except for genes *dat* and *lhkA,* which clearly associated CLIP98 with lineage II (not shown). Close inspection of the sequence alignment showed that a large proportion of nucleotide changes that distinguished CLIP98 from lineage II strains were clustered in a small number of short segments and corresponded to nucleotide bases also observed in *L. innocua* strains.

The phylogenetic relationships within lineage I (**Figure 3B**) suggest that serotype 4b is monophyletic, since all strains of this serotype formed a unique branch. Differently, serotype 1/2b is paraphyletic, as it was encountered in two distinct branches, one of which is branching off early in the history of lineage I. Within lineage II, serotype 1/2a was paraphyletic, whereas 1/2c was monophyletic (**Figure 3C**). Notably, the sequenced strain EGDe (1/2a) appears to branch off just before the evolutionary change from 1/2a to 1/2c.

### Evolution of *inlA* coding sequences: local recombination and convergence of truncated forms in defined clones

**Figure 4:**
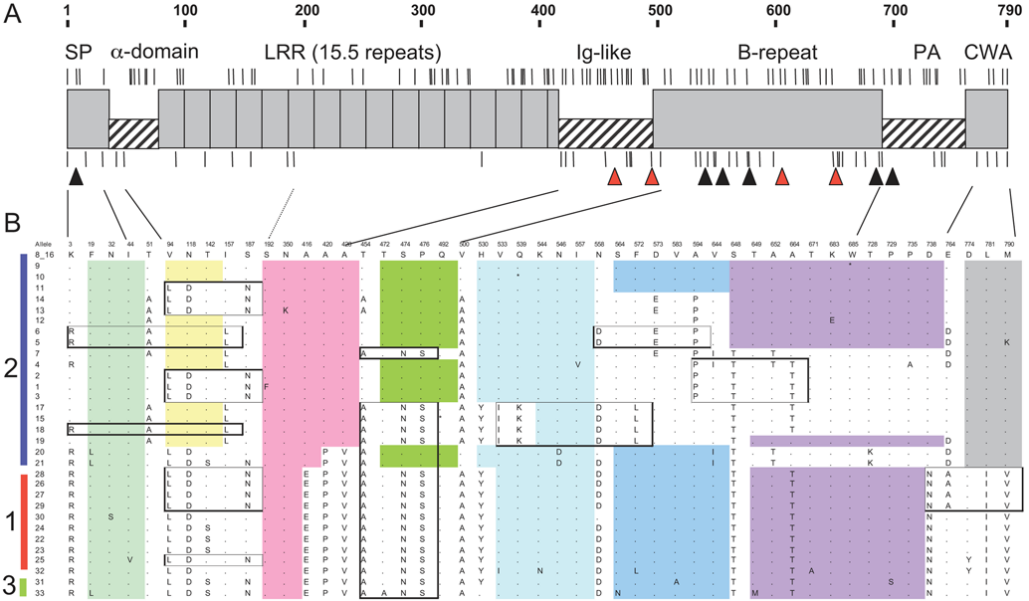
InlA polymorphisms. A) Distribution of the polymorphisms along the 2,400 nt of gene *inlA* in the 33 distinct *inlA* alleles encountered. The scale above the graph is in amino-acids (AA). InlA functional domains are represented as distinct blocks: signal peptide (SP), alpha-domain linker, leucine-rich repeats (LRR), Ig-like, B-repeat, Pre-anchor (PA) and cell wall anchor (CWA). Vertical bars above these blocks, correspond to synonymous nucleotide polymorphisms; below these blocks, non-synonymous polymorphisms resulting in amino-acid changes. Note that the LRR domain, especially repeats 7 to 15, is highly conserved. Triangles indicate the position of premature stop codons (PMSC) observed in this study and previous reports; black triangles: PMSCs observed in clonal complex CC9; red: PMSCs in other clones; see Table 4 for details. B) Deduced amino-acid polymorphisms in InlA. Lineages in which the *inlA* alleles were found are indicated on the left (1, 2 or 3). Blocks of amino-acids that are identical to the sequence in reference strain EGDe (allele 8) are color-shaded. Note the mosaic pattern, with blocks of polymorphisms shared between distinct groups of alleles when scrolling along the sequence.

The 2,400-nt coding sequence of virulence factor InlA showed 162 (6.7%) polymorphic sites and 33 alleles undergoing a distinctive pattern of evolution (**Figure 4**). First, in contrast to housekeeping genes, phylogenetic analysis of *inlA* sequences revealed a conspicuous pattern of intragenic homologous recombination. Visual inspection of the distribution of polymorphic sites in *inlA* revealed a mosaic structure (**Figure 4**), with several regions having estimated recombination rates one order of magnitude higher (ρ≥0.03) than for baseline regions (ρ≈0.003). Thus, each *L. monocytogenes inlA* sequence represents a composite assembly of short sequences with distinct evolutionary history, likely the result of multiple horizontal gene transfer events. Notably, in no case did we find fully- or nearly identical *inlA* sequences in unrelated STs, showing that horizontal transfer of entire *inlA* alleles is infrequent or non-existing, and that the entire *inlA* coding sequence is clone-specific. The short-term and long-term impacts of localized recombination in the *inlA* sequence were contrasted. Over the short term, *inlA* sequences clearly evolved more rapidly than housekeeping genes. For example, *inlA* sequences within lineage II evolved more than twice as fast (c = 2.13, r = 18%) as MLST genes. In contrast, over the long term, *inlA* sequence divergence was restricted, as housekeeping genes were on average more divergent between the three major lineages than are *inlA* sequences (e.g., 4.8% and 1.3%, respectively, between lineages I and III strains). This constraint on *inlA* sequence divergence resulted in the lack of phylogenetic demarcation of lineages I and III (**Figure S1**), contrasting sharply with housekeeping genes-based phylogeny (**Figures 2** **and** **3**). Thus, import of sequence stretches from other clones accelerates diversification of clones, while homogenizing *inlA* sequences among distantly related strains.

**Table 4:** Premature stop codons identified in internalin gene *inlA* of *Listeria monocytogenes* isolates.

AA position of stop codon	AA position of mutation	Nt position of mutation	*inlA* allele	ST (CC)	Isolates or mutation name	Reference
8	2	6 (1 nt del.→frameshift)	Id. to inlA-8 except for pos. 6	ST9 (CC9)	3 strains	Orsi et al., 2006
460	513	1,539 (1 nt del.→frameshift)	undefined	lineage II	NV8	Rousseaux et al., 2004
492	492	1,474 (C→T)	inlA-15	ST121	LM71322, LM74705	This study
492	492	1,474 (C→T)	Id. to inlA-7 (over pos. 859–1,591) except pos. 1,474	ST20 ?	H1	Olier et al. 2002; Olier 200et al., 2003
519	513	1,539 (1 nt del.→frameshift)	1 SNP and 1 del. to inlA-8 (over pos. 859–1,591)	ST9 (CC9)	NV7	Rousseaux et al., 2004
539	539	1,615 (C→T)	inlA-10	ST9	LM57179	This study
577	545	1,636 (1 nt del.→frameshift)	inlA-16	ST9	LM6186, LM70179, LM73771, LM95474	This study
577	546	1,637 (1 nt del.→frameshift)	undefined	ST9 (CC9)	LO28	Jonquieres et al., 1998
606	606	1,818 (T→A)	Id. To inlA-29 (over pos. 1,642 to 2,347) except pos. 1,818)	ST5 or close to ST5	Mutation type 1	Nightingale et al., 2005
656	656	1,966 (C→T)	3 SNPs to inlA-29 (over pos. 1,642 to 2,347)	ST5 or close to ST5	Mutation type 2	Nightingale et al., 2005
685	685	2,054 (G→A)	inlA-9	ST9	LM15839, LM77097, LM77571, LM86391, LM93096	This study
685	685	2,054 (G→A)	Id. to inlA-9 (over pos. 859–2,189)	ST9 (CC9)	NV5	Rousseaux et al., 2004
700	700	2,100 (C→G)	Id. to inlA-8 (over pos. 1,642 to 2,347) except pos. 2,100	ST9 (CC9)	Mutation type 3	Nightingale et al., 2005

ST: sequence type; CC: clonal complex; pos.: position; Id.: identical; nt: nucleotide; del. Deleted; SNP: single nucleotide polymorphism.

Second, when the entire length of *inlA* was considered, purifying selection against amino-acid changes appeared more relaxed than for housekeeping genes, with a Ka/Ks ratio for *inlA* (0.094) higher than for the seven concatenated MLST genes (0.039 for the same 157 isolates). However, the distribution of ω (the Bayesian estimate of the rate of synonymous vs. non-synonymous substitutions) along the sequence was heterogeneous, with a highly constrained LRR-region (ω≈0.04) and moderately constrained Ig-like and B-repeats regions, with peak values of 0.5. It is worth mentioning that no single stretch of the molecule displayed a significant signature of positive selection, a result that contradicts previous analyses in which recombination was not incorporated [59]. All but one amino-acid changes found in the LRR domain were located in repeats 1 to 6 (**Figure 4**), suggesting a stronger constraint on repeats 7 to 15, which are more extensively involved in interactions with E-cadherin [60].

Truncated forms of InlA have been described and associated with reduced virulence [5], [16], [59], [61]–[64]. We found four distinct *inlA* alleles that had premature stop codons (PMSC) at positions 492, 539, 577 and 685 (**Figures 1** **and** **4** **;** **Table 4**). Remarkably, three of these four PMSCs occurred in isolates that belonged to CC9 (**Figure 1**). In addition, out of eight previously reported *inlA* sequences leading to truncated forms, five were identical or nearly identical (<2 SNPs) to the *inlA* sequences that are specific of clone CC9, strongly suggesting that they first occurred in isolates of clone CC9 as well. The remaining PMSC (at codon 492) was observed in ST121 (1/2a), and three other PMSCs from previous reports were also observed in *inlA* alleles unrelated to those in CC9 (**Table 4**).

## Discussion

The phylogenetic structure of *L. monocytogenes* was investigated to provide a framework for the evolutionary history, epidemiology and virulence of this model pathogen. We based our analysis on an update of the MLST scheme proposed by Salcedo et al. [14]. Alternative sets of genes were used previously [15], [65]–[67], but were either not extensively validated [65], biased towards high levels of nucleotide diversity between two particular lineages [66] or based at least in part on virulence genes [15],[22],[67]. Virulence-associated genes generally provide improved discrimination among strains, but may reflect ecological adaptation and selection. In contrast, housekeeping genes are considered more appropriate to obtain an unbiased view of the population structure, as their polymorphisms can be considered nearly neutral and are less subject to horizontal transfer.

### Clonal structure of *L. monocytogenes*

A majority of *L. monocytogenes* isolates belonged to limited number of major clones. Although these were defined based on allelic profiles, the same groupings were obtained by MST analysis based on nucleotide sequences (not shown), with the exception of the three STs (ST35, ST15 and ST74) inferred to derive by a recombination event that changed more than two SNPs. Hence, no significant loss of information was incurred by collapsing nucleotide sequence information into allelic profiles, as expected given limited amounts of recombination. Interestingly, the major clones were almost always separated from other strains by at least three allelic mismatches, indicating ancient divergence. Hence, a relaxed criterion (e.g., two allelic mismatches) would have little impact on the assignment of *L. monocytogenes* isolates to particular clones. Given the neat delineation of the major clones and the fact that they account for a large proportion of clinical *L. monocytogenes* isolates, we propose that these genetic entities represent reference units for future studies on strain virulence, ecology or epidemiology.

For global population biology and international surveillance purposes, a definitive strain typing scheme is greatly needed [18]. The present MLST data represent a unifying language on clone characterization in *L. monocytogenes* and are freely available for comparison at www.pasteur.fr/mlst. Other sequence-based strain characterization methods have been developed [15]–[17],[34]. Future determination of the relative power of MLST and these methods for discrimination among *L. monocytogenes* strains, as well as establishment of the correspondence among the sequence types they define, is required for optimal communication and will provide *Listeria* specialists with a choice of methods that may be suited for distinct purposes (e.g., fine-scale epidemiology versus long-term population biology).

Although our strain collection was not designed to address the important question of ecological or epidemiological differences among *L. monocytogenes* clones, we found different distributions of serotypes and clones among animal, environmental and clinical isolates that are consistent with previous reports [26],[27]; for example, serotypes 4b clones mostly included isolates from clinical or food sources, whereas they are rare among isolates from animals (4 out of 21) or the environment (none out of 9). However, further MLST studies should be performed with ecologically representative collections of isolates. Likewise, our initial temporal analysis of maternal-fetal isolates suggests the existence of temporal shifts in prevalence of clones over relatively short periods of time (19 years), but a larger longitudinal survey is needed to provide a clearer picture of the temporal dynamics of these clones. Finally, the disclosed diversity is based largely on isolates from France, and a worldwide collection may therefore reveal additional diversity. However, it is noteworthy that many reference strains from outbreaks in other countries and continents belonged to clones defined by French isolates (**Figure 1**). It is also important to remember that *L. monocytogenes* is an environmental saprophyte, which does not need to infect animals for survival and propagation. The diversity of clinical isolates may thus only represent a particular subset of the entire diversity of this species.

### Evolution of serotypes

The evolutionary relationships of serotypes within the major lineages have not been previously defined, although this knowledge is particularly important for correct interpretation of serotyping data. We found that all serotype 4b strains belonged to a unique branch, consistent with early MLEE data [3] and recent sequence data [16]. This result indicates that serotype 4b appeared only once during the evolution of lineage I. Hence, the apparent increased potential of serotype 4b strains to cause outbreaks may be explained by genetic characteristics that evolved ancestrally, before the diversification of serotype 4b into several clones that appear highly successful among clinical isolates. The fact that the 4b branch is nested within a larger diversity made of 1/2b strains suggests that serotype 1/2b is more ancestral than 4b, and possibly represents the ancestral serotype of lineage I. Likewise, within lineage II, paraphyletic serotype 1/2a clearly stands as the most likely ancestor of lineage II, with serotype 1/2c having evolved more recently (**Figure 3C**). As a consequence, our results contradict the DNA array-derived hypothesis that serotype 1/2c represents an ancestral state [28]. Notably, strain EGDe (1/2a) appears to branch off from the ancestor of CC9 1/2c strains just before the evolutionary shift from 1/2a to 1/2c. Hence, strain EGDe may represent a genomic state close to the evolutionary link between 1/2a and 1/2c strains. While exhibiting an antigenic structure that retained ancestral characteristics, its full genomic content [57] is likely to be more related to that of 1/2c strains than to most other 1/2a strains, consistent with DNA array data [28],[41].

Groups of distinct serotypes observed in isolates with the same ST or CC reflect relatively recent evolution of antigenic structures. Under our evolutionary scenario (**Figures 1** **and** **2**), serotypes 7 and 3b derived from serotype 1/2b in the branch corresponding to clone 3, whereas serotypes 4d and 4e each derived at least twice from distinct 4b ancestors, as also proposed recently [16]. Likewise, serotype 3c may be derived from 1/2c strains (although the reverse, from 3c to 1/2c, cannot be excluded). All these evolutionary changes in serotype involve modification of the somatic antigens [68]. In contrast, H (flagellar) antigens appear stable over evolutionary time, as we inferred a single within-lineage change of the H antigen, namely from antigen A to antigen D, corresponding to the evolution of 1/2c [antigenic formula I,II,(III):B,D] from 1/2a [I,II,(III):A,B] [68]. Knowledge and subsequent characterization of the genetic determinants of somatic and flagellar antigenic structures [69],[70] will provide molecular details on serotype evolution.

### Phylogenetic structure of *L. monocytogenes* and recombination

The distinct phylogenetic methods used herein consistently identified three major lineages of *L. monocytogenes* strains, in agreement with a wide set of previous studies based on alternative markers [25]–[27]. The only exception was isolate CLIP98, placed at the tip of a long branch with weak relatedness to any lineage. However, we suggest that CLIP98 does not represent the first disclosed member of a fourth *L. monocytogenes* lineage. Instead, the fact that two genes of CLIP98 were typical of lineage II, whereas numerous polymorphisms in the other five genes were shared with *L. innocua*, suggests that CLIP98 could have a ‘hybrid’ genome derived from lineage II, but which has received from *L. innocua* donors enough recombined fragments to now appear poorly affiliated with its ancestral lineage. Because putative recombined segments are very small and often consist of only two or three SNPs, these imports were overlooked by ClonalFrame. As three nucleotides of CLIP98 (between positions *cat* 364–368) were also uniquely shared with lineage III strains, CLIP98 may correspond to a recombinant strain with multiple ancestries, possibly due to an increased capacity for incorporation of foreign DNA. Further genomic characterization of this isolate is needed to clarify these aspects.

*L. monocytogenes* lineages differed from *L. innocua*, their closest relative, by 11% of nucleotide positions on average. This is consistent with the monophyly of *L. monocytogenes* and does not support the hypothesis of a descent of *L. innocua* as a whole from *L. monocytogenes* [28]. Amounts of diversity within the three lineages were 0.33%, 0.61% and 1.25%, respectively, consistent with data obtained by others [22],[27]. In contrast, the nucleotide divergence was 4.99% between lineages I and II, 5.3% between lineages I and III, and 7.57% between lineages II and III. Notably, there was not a single common allele among the three lineages, as well as between them and *L. innocua*, whereas strains within a particular lineage generally shared at least one allele. Hence, consistent with DNA hybridization data [71], the three major lineages correspond to clearly demarcated sequence clusters that fulfill the separateness criteria and divergence levels used in other bacterial groups to distinguish species [72]–[75]. However, as noted earlier [27], the issue of taxonomic revision of *L. monocytogenes* needs careful evaluation. In particular, improved sampling (especially from diversity-rich sources such as the environment) would be necessary to challenge the neat demarcation among lineages and characterize their ecology.

The rate of homologous recombination within bacterial species can differ widely from one species to another [76],[77] and has a profound impact on the validity of phylogenetic analyses [78], on the evolutionary stability of genotypes [79], on biological features such as virulence [35],[80] and on interpretation of typing data [20]. Previous reports have indicated that *L. monocytogenes* undergoes low levels of recombination among housekeeping genes [3],[14]. Here, we quantified the relative impact of recombination and mutation at various levels of phylogenetic depth and found similar estimates with independent approaches. To our knowledge, *L. monocytogenes* is one of the bacterial species with the lowest rate of recombination [50],[77],[81]. Highly restricted levels of recombination were disclosed in all three major lineages, contrasting with a previous proposal that rates of recombination vary among lineages [66]. Full genome sequencing [57] suggested that competence genes are present in *L. monocytogenes* EDGe (CC9), but the regulatory genes for their expression have not been identified, and a noncoding RNA (RliE) may regulate negatively the *L. monocytogenes* orthologs required for competence in *Bacillus subtilis* [82]. Yet, evidence that *L. monocytogenes* retained the ability for localized recombination is clearly provided by the *inlA* gene encoding InlA, in agreement with previous reports [22],[27],[59]. For this gene, recombination events may contribute to the acquisition by the recipient strain of a selective advantage, for example by escaping the immune response while retaining the ability to interact with its receptor. Such exchange could possibly occur in the intestinal lumen, in which multiple *L. monocytogenes* strains may coexist. Hence, rapid diversification of *inlA* sequences contrasts with the inferred high stability of clonal backgrounds defined by housekeeping genes. This illustrates how the phylogenetic structure based on MLST genes provides a scaffold, which sheds light onto the evolution of individual genes exposed to selective pressures, such as virulence genes.

### InlA evolution

Currently, the ecological significance of loss of a full-length InlA is not understood, and the clonal background in which these forms evolve have not been defined precisely. Little is known about the ecology of *L. monocytogenes* clones, but a realistic scenario is that different clonal families might be adapted to different niches, and their occurrence as mammalian pathogens may be of limited significance for their evolutionary success in the long term. Among the four alleles of gene *inlA* identified among ST9 isolates, the one corresponding to the non-truncated form (*inlA*-8) can be inferred to be ancestral, as the three *inlA* alleles corresponding to truncated forms (inlA-9, inlA-10 and inlA-16) differed by only one mutation from inlA-8, whereas they differed by two mutations from each other (**Table 4**). In addition, *inlA*-8 was also found in strain EGDe (ST35), which was inferred to branch off before diversification of other CC9 members (**Figure 3**). It is intriguing that InlA, an important bacterial factor for host colonization, was repeatedly lost by convergent evolution in the genetically homogeneous 1/2c ST9 genotype. Such a pattern can be explained either by a relaxed selective constraint on maintaining InlA function, or by a selective advantage provided by the loss of a functional InlA protein, in the ecological niche occupied by members of ST9. Determining the natural habitat of ST9 may provide clues as to why the expression of a virulence trait may in fact turn out to be disadvantageous in particular environments.

## Supporting Information

Figure S1 inlA phylogeny. Neighbor-joining tree of the 33 inlA alleles (midpoint rooting). Alleles resulting in truncated forms of the protein InlA are indicated by a delta letter followed by the position of the stop codon. Isolates in which the alleles were observed are indicated in Table S1. The lineages in which the alleles were observed are indicated by colored vertical bars on the right. Note the lack of separation of lineages I and III, as opposed to the results obtained with housekeeping genes (Figures 2 and 3).
(0.25 MB EPS)

Table S1 Strains
(0.09 MB XLS)

Table S2 RDP analysis
(0.03 MB XLS)
